# Use of N-butyl cyanoacrylate in the successful transcatheter arterial embolization of an arteriovenous fistula caused by blunt pelvic fracture: A case report and review of literature

**DOI:** 10.1097/MD.0000000000024215

**Published:** 2021-01-08

**Authors:** Hye Soo Cho, Yook Kim, Jisun Lee, Kyung Sik Yi, Chi-Hoon Choi

**Affiliations:** Department of Radiology, Chungbuk National University Hospital, Cheongju, Korea.

**Keywords:** and embolization, arteriovenous fistulas, blunt pelvic bone fracture

## Abstract

**Rationale:**

Traumatic arteriovenous fistulas (AVFs) of the pelvis are uncommon and present with a variety of clinical manifestations; their detection may be difficult. An endovascular approach is usually the first choice of treatment, because surgical intervention is complicated due to the location of the lesions.

**Patient concerns:**

A 68-year-old man was admitted with severe pelvic pain following a fall.

**Diagnosis:**

A pelvic bone fracture (Young and Burgess Classification, lateral compression type II) was revealed on pelvic computed tomography (CT), while a pelvic sidewall hematoma, unaccompanied by any vascular injury, was detected on multidetector CT.

**Interventions:**

Pelvic angiography revealed an AVF between the internal iliac artery and vein, which was undetected by MDCT. The AVF was successfully treated using transcatheter arterial embolization (TAE) with n-butyl cyanoacrylate (NBCA).

**Outcomes:**

The patient recovered well and was discharged 4 weeks later. No complications were noted at the 8-month follow-up.

**Lessons:**

AVF may occur as a complication of blunt pelvic bone fracture. A high index of suspicion, angiography, and prompt diagnosis resulted in the successful management of our patient who presented with risk factors. Furthermore, TAE using NBCA enables a minimally invasive and effective treatment of traumatic pelvic AVF.

## 1 Introduction

Pelvic bone fractures associated with vascular injuries can result in a mortality rate that is as high as 50%.^[1]^ Transcatheter arterial embolization (TAE) is effective for the safe and efficient management of hemorrhages arising from pelvic trauma.^[2]^ Arterial injuries following pelvic trauma include occlusion, dissection, pseudoaneurysm, and arteriovenous fistulas (AVFs).^[3,4]^ Traumatic AVF is an abnormal and rare connection between an artery and an adjacent vein, resulting from a simultaneous damage to the two.^[3]^ It presents with a variety of clinical symptoms and is often difficult to detect^[4]^; if undiagnosed, the left-to-right shunt may cause progressive dilatation of the involved veins and may lead to pulsatile venous or lymphatic complications.^[5,6]^ Over time, heart failure may occur. To date, few cases involving traumatic pelvic AVFs have been reported.^[5–8]^ Herein, we report a case with pelvic AVF caused by blunt trauma that was successfully treated by TAE with *n*-butyl cyanoacrylate (NBCA). To our knowledge, this is the first case report on the use of NBCA as an embolic material for treating pelvic AVF. The study design was approved by the Chungbuk National University Hospital Institutional Review Board, and the written informed consent was obtained from the patient for publication of this case report and accompanying image.

## 2 Case presentation

**Figure 1:**
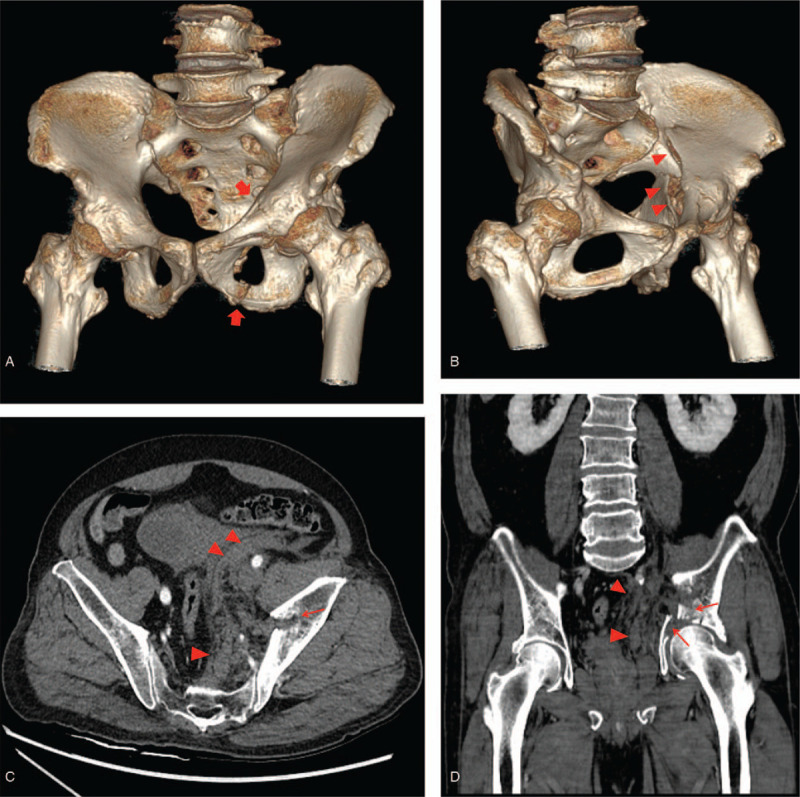
A 64-year-old man was admitted to our trauma center after falling from a height of 2 m. (A and B) A volume-rendered 3D-reconstructed image shows multiple fractures involving the left superior and inferior rami (red arrow) and a comminuted left iliac bone fracture (red arrow head). The suggested fracture type is the Young and Burgess Classification of lateral compression type II. (C and D) Axial and coronal CT scans demonstrate a comminuted left iliac fracture (thin red arrow) with a pelvic side wall hematoma (red arrow head); no evidence of trauma-related vascular injury is observed.

**Figure 2:**
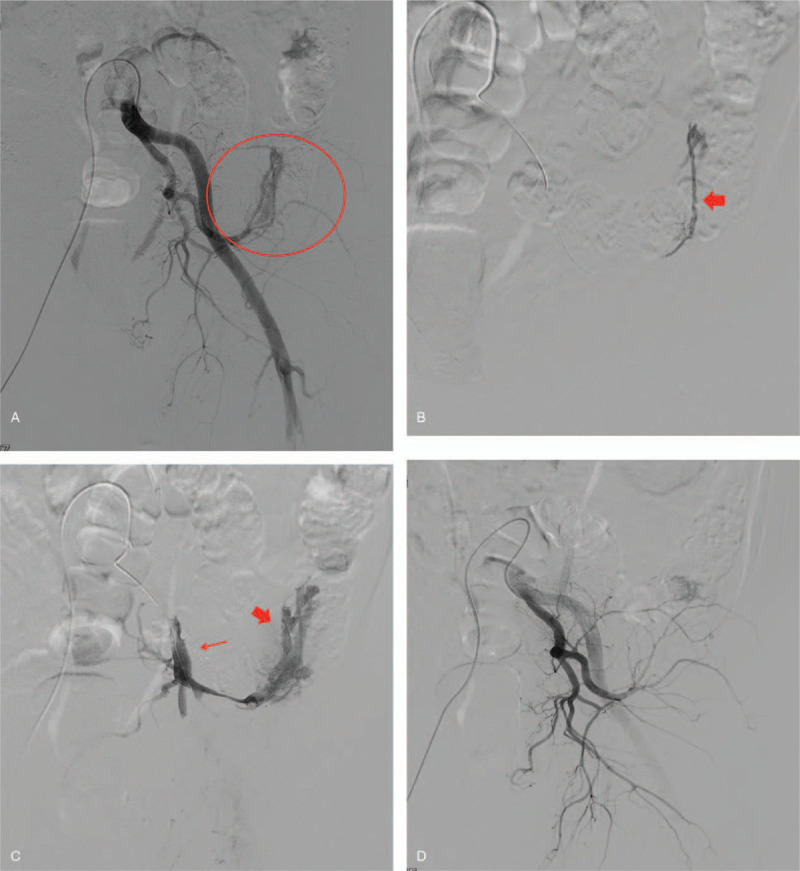
A 64-year-old man admitted to our trauma center after falling from a height of 2 m. (A) Left common iliac artery angiography, performed via a right femoral approach, reveals an abnormal vascular structure including irregularity and caliber changes, suggesting a vascular injury in the left lateral side of the pelvis (red circle). (B) A selective angiography (using a microcatheter) of the culprit artery, that is, the distal branch of the obturator artery, reveals diffuse luminal irregularity of the feeding artery (read arrow) (C) with a dilated vein (thick red arrow) and early filling of the left lumbar vein (thin red arrow). These findings indicate AVF. (D) Postembolization angiography demonstrates successful hemostasis with exclusion of the AVF.

A 64-year-old man was admitted to the emergency department after falling from a height of 2 m. He had a history of cardiovascular disease and was on anticoagulant drugs for the past 10 years. On admission, his blood pressure, pulse, and hemoglobin level were 97/63 mmHg, 160 beats/min, and 10.3 g/dL, respectively. Although his vital signs stabilized after fluid resuscitation, he complained of pain in his left hip. Subsequent physical examination revealed diffuse tenderness in the left hip and the pelvis. Pelvic computed tomography (CT) revealed a pelvic bone fracture (Young and Burgess Classification of Lateral compression type II) (Fig. 1A and B), while multidetector CT revealed a left pelvic sidewall hematoma that was not accompanied by any vascular injury (Fig. 1C and D). Although the patient was hemodynamically stable and there was no evidence of any vascular injury on the initial CT image, angiography was undertaken as a diagnostic and therapeutic option, considering the patient's age and medical history and the high-risk pelvic fracture pattern. Left common iliac artery angiography, performed via a right femoral approach, revealed an abnormal vascular structure with irregularity and caliber changes, indicating a vascular injury in the left lateral side of the pelvis (Fig. 2A). A selective angiography of the distal branch of the obturator artery (i.e., the culprit artery) revealed diffuse luminal irregularity of the feeding artery with a dilated vein and early filling of the left lumbar vein, suggesting an AVF (Fig. 2B and C). To use microcolis as the embolic material, we attempted to advance the microcatheter tip distal to the injured artery for avoiding a technical failure or any procedure-related complication. However, due to vulnerable of the arterial wall caused by pelvic trauma, the microcatheter tip was actually away from the distal portion of the injured artery. Subsequently, we resorted to using *n*-butyl cyanoacrylate (NBCA) as the embolic material. A 1:2 mixture of NBCA and iodized oil was injected, followed by contrast medium injection that demonstrated the disappearance of the AVF (Fig. 2D). The postoperative course was otherwise unremarkable and the patient was discharged 1 month after the procedure. No complications were noted at the 8-month follow-up assessment.

## 3 Discussion

AVF is an abnormal connection between an artery and an adjacent vein that does not pass via the capillary network.^[1–3]^ Although its incidence in pelvic blunt trauma is very low, early identification and appropriate treatment is critical because these carry a high risk of complications.^[5–7]^ On reviewing existing literature (in English), to the best our knowledge, only 3 cases of angiography-confirmed AVFs in blunt traumatic pelvic bone fractures have been reported (Table 1).^[5,6,9]^ The time interval between pelvic injury and angiography diagnosis ranged from 4 hours to 20 years. The initial treatment for pelvic AVF included an open surgery in 1 patient^[9]^ and endovascular therapy in the other 2 patients.^[5,10]^ Rebleeding occurred in 2 patients, which was successfully treated with surgery. In our case, pelvic AVF was confirmed by angiography 3 hours after the injury and was successfully treated by embolization.

**Table 1:** Review of pelvic injuries associated with traumatic arteriovenous fistulas.

Ref.	Year	Sex/Age, y	Trauma type	Interval^∗^	Pelvic facture type^†^	CT	Angiography	Embolic material	Technical success	Clinical success	Remark
Present case	2020	M/64	Fall	1 h	LC type II	Left pelvic sidewall hematoma unaccompanied by vascular injury	AVF between the distal branch of the OA and the left lumbar vein	NBCA	Yes	Yes	
Vinu et al^[5]^	2018	M/84	MVC	4 h	Tile B	Blush of contrast from the distal branch of the right IIA	AVF between the SGA and SGV	Embosphere and microcoil	Yes	No	Second embolization due to rebleeding^‡^
Maroon et al^[9]^	1988	M/26	MVC	1.5 yr	Multiple pelvic fractures	NA	A large AVF between the left LSA and vein	Open surgery	Yes	Yes	
Claude et al^[10]^	1988	M/63	MVC	13 yr	Pelvic fracture	NA	A large AVF between the right SGA and SGV	Balloon catheter	No	No	Open surgery for the control of AVF ^§^

Currently, AVF can be diagnosed using duplex and color Doppler ultrasound, CT, and angiography.^[3,4]^ CT, which is now readily available in many emergency departments, has shown a high sensitivity (90–100%) and specificity (98–100%) for the detection of traumatic vascular lesions, including AVF.^[3]^ In patients with a traumatic AVF, early filling of the pelvis with the contrast material is often the only finding on an MDCT scan.^[4]^ However, CT maybe limited in locating the AVF, especially because fistulas may form weeks to years after an injury and signs associated with vascular injury, such as pseudoaneurysm, may be absent.^[3–6]^ Although invasive, angiography is the best diagnostic tool for detecting vascular pathologies, especially hemorrhages.^[3,5–8]^ It can continuously detect the anatomical source of the bleeding and thus prevents the theoretical risk of missing a delayed extravasation and guides endovascular treatment. The basic indication for angiography is the suspicion of an injured pelvic artery, that is, active extravasation of contrast medium observed in a CT scan.^[1–3]^ When hemorrhage is not observed in the initial CT scan, the decision to perform an angiography depends on several factors, including the patient's clinical scenario, vital signs, and continued need for resuscitation; angiographer's availability; and physician's experience.^[2–4,6]^ Advanced age (above 60 years) is an independent predictor of vascular injury and indication for angiography, regardless of hemodynamic stability.^[1,2]^ Long-term anticoagulant usage for cardiovascular diseases, as in this patient, may also necessitate an early intervention.^[1,2]^ Although basing the decision to perform angiography on the pelvic fracture type alone is not recommended, Vaidya et al^[1]^ found that Young and Burgess fracture type LC, followed by APC, are the most concerning fracture patterns to have vascular injuries requiring embolization. Therefore, it is important that physicians recognize these patterns in the absence of active extravasation in the initial CT scan, as these may suggest the culprit vessel. In our case, although there was no evidence of vascular injuries related to pelvic bone trauma on the initial CT scan, considering the patient's age and history of anticoagulant usage and the high-risk pelvic bone fracture pattern, a diagnostic angiography was performed. Subsequently, an AVF originating from an internal iliac artery branch could be identified. Thus, angiography would be the most useful diagnostic modality in high-risk pelvic trauma patients.

A wide spectrum of therapies for traumatic AVF, including open surgery and TAE, has been proposed. Case reports published in the last decade indicate that an increasing number of physicians have chosen to undertake TAE for treating hemorrhages caused by pelvic trauma; retrospective reviews have revealed that the clinical success rate is greater than 90%, with a 4% complication rate.^[1,2]^ Compared with surgery, TAE is a safer and a more effective treatment option, offering low procedure-related complication rates, avoidance of surgical risks, and shorter hospitalization.^[1]^ The type and method of delivery of the embolic agent should be decided on the basis of angiographic findings of the vessel injury. In case of AVFs caused by trauma, coils may be the ideal agent as they allow precise embolization^[1,3–6]^; however, there is a risk of coil migration into the venous system and thereafter centrally. Furthermore, delivering the coil to the bleeding site is not always possible due to small vessel size or tortuous anatomy. Another treatment option includes the exclusion of the fistula with NBCA. NBCA is widely used for controlling active bleeding following trauma.^[7]^ It rapidly polymerizes with the blood and due to its high penetrability secondary to its liquid nature, can obliterate small distal vessels. Therefore, it is advantageous for massive hemorrhages that requires urgent hemostasis.^[8,9]^ In our case, TAE was performed using NBCA, and a technically successful embolization was achieved. The patient was discharged without recurrent bleeding.

We recommend that the following points be considered when treating patients with pelvic bone fractures: It is essential that trauma surgeons or intervention radiologists be familiar with the patient's medical history and previous radiological scans in order to ensure that angiography are undertaken appropriately at the correct time. This will allow them to decide immediately whether treatment is necessary or not.

## 4 Conclusion

AVF originating from the internal iliac artery may arise as a complication of blunt pelvic bone trauma. The patient's medical drug history and age, in addition to the pelvic bone fracture type, is helpful in predicting the need for angiography even when there is no definite evidence of hemorrhage on the initial CT scan. Minimally invasive TAE is a safe and efficient treatment for pelvic AVF. When the injured artery is insufficiently selected by the microcatheter, NBCA could be considered as a useful, permanent embolic material in the embolization of pelvic AVF.

## Author contributions

**Conceptualization:** Yook Kim.

**Data curation:** Hye Soo Cho.

**Investigation:** Hye Soo Cho.

**Visualization:** Hye Soo Cho, Yook Kim.

**Writing – original draft:** Hye Soo Cho, Yook Kim.

**Writing – review & editing:** Hye Soo Cho, Yook Kim, Jisun Lee, Kyung Sik Yi, Chi-Hoon Choi.
